# 2437. The Impact of Healthcare Associated Infections on Costs and Lengths of Stay 2019-2023

**DOI:** 10.1093/ofid/ofad500.2056

**Published:** 2023-11-27

**Authors:** Jennifer L Sentiff, Vamsi Yenugadhati, Courtney Spitz, Shannon Glassman, Matthew Phillips, Stephany Frey, Emil P Lesho

**Affiliations:** Rochester Regional Health, Rochester, New York; Rochester Regional Health, Rochester, New York; Rochester Regional Health, Rochester, New York; Rochester Regional Health, Rochester, New York; Rochester Regional Health, Rochester, New York; Rochester Regional Health, Rochester, New York; Rochester Regional Health, Rochester, New York

## Abstract

**Background:**

As 85% of hospitals in the U.S. face unsustainable operating margins, extraordinary labor costs (LC) from nursing shortages, and have under resourced infection prevention departments, updated assessments of the costs of healthcare associated infections (HAI) are needed. However, most published cost analyses are either more than a decade old, or did not adjust for timing of infection. We sought to determine excess lengths of stay (LOS) and LC associated with HAI during the COVID-19 era, while adjusting for timing of infection.

**Figure:**
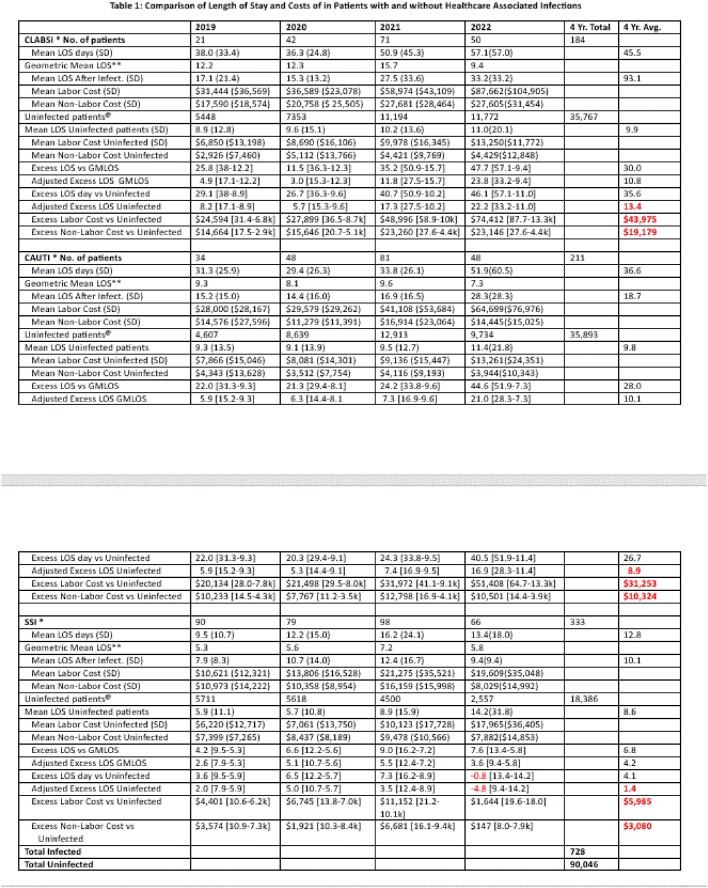

Timing of Infection Onset In Patients with an HAI

**Figure:**
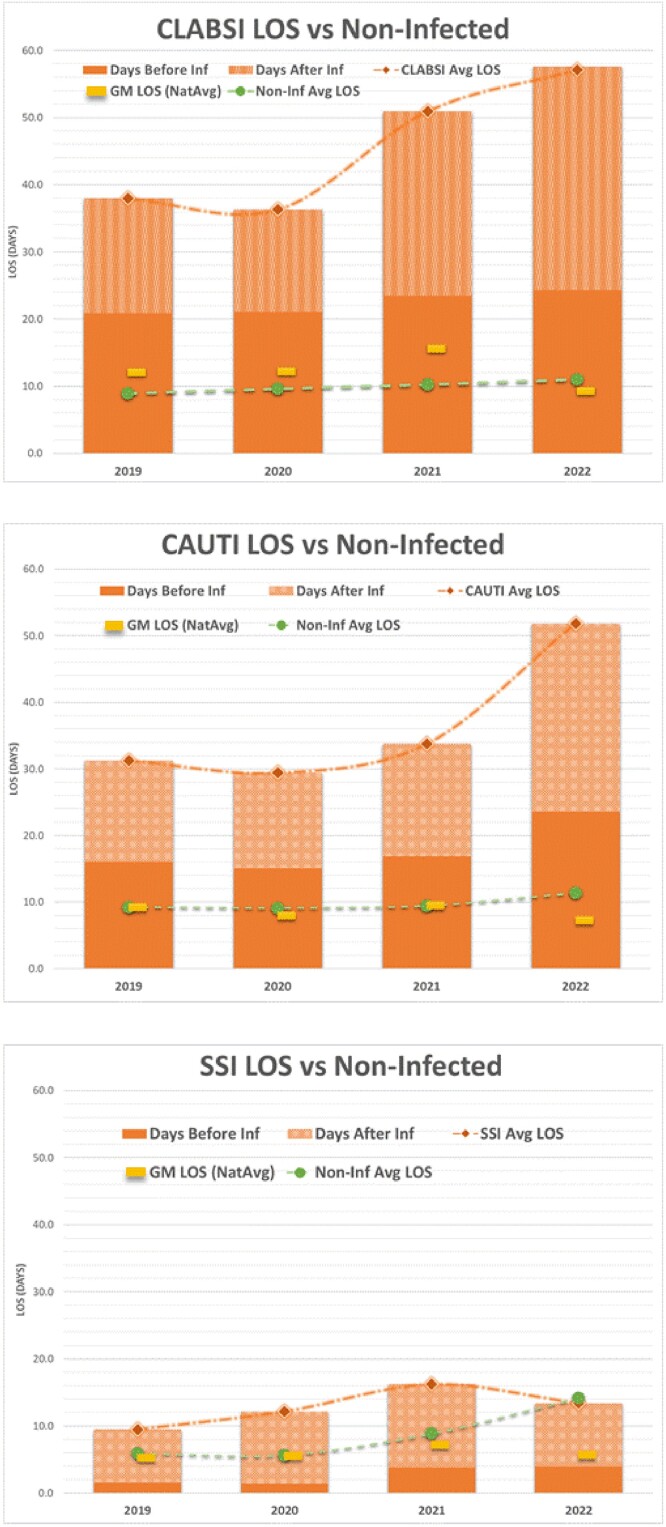

Legend: dark orange = days before infection; light orange = days after infection; yellow bar = geometric mean national average length of stay of uninfected patients with the same diagnosis code; green circle = mean length of stay of local uninfected controls

**Methods:**

All inpatients in an academic tertiary care center and 4 community hospitals in Western NY from 01-01-19 to 12-31-22 were included. Using nationally standardized definitions of catheter-associated urinary tract (CAUTI), central line associated bloodstream (CLABSI), and surgical site infections (SSI), 728 HAIs were identified and compared to patients during the same encounter with the same diagnosis codes (DRG) without an HAI (n= 90,046). Only days after infection onset were used to calculate excess LOS and cost.

**Results:**

The mean yearly standardized infection ratios (SIRs), as defined by the National Healthcare Safety Network, were 0.89, 0.98, and 1.14 for CLABSI, CAUTI and SSI, respectively. Patients with an HAI had significantly higher LC and longer post-infection LOS (all p-values < 0.005) compared to a national benchmark and their DRG-matched HAI-negative counterparts. Per admission, the average excess labor cost and excess LOS were $43,975, 13.4 days for CLABSI; $31,253, 8.9 days for CAUTI; and $5,985, 1.4 days for SSI (Table). The 4-year total was $23.4 M, and 4810 excess days. 100% of CLABSI and CAUTI occurred after the 13th day, and the pre and post-infection LOS were similar. SSI occurred early in admission, and the post-infection LOS was longer than the pre-infection LOS (Figure). Re-admission occurred for 83% of SSI. For CLABSI and CAUTI excess LOS and costs increased 178% over the study period.

**Conclusion:**

These findings suggest that for CLABSI and CAUTI, minimizing pre-infection LOS can provide a greater economic and safety impact than focusing entirely on preventing the infection. However, for SSI, focusing on preventing the infection may provide the greatest benefit. Other hospitals can adjust these data based on their individual SIRs.

**Disclosures:**

**All Authors**: No reported disclosures

